# Conditional variable importance for random forests

**DOI:** 10.1186/1471-2105-9-307

**Published:** 2008-07-11

**Authors:** Carolin Strobl, Anne-Laure Boulesteix, Thomas Kneib, Thomas Augustin, Achim Zeileis

**Affiliations:** 1 Department of Statistics, Ludwig-Maximilians-Universität Munchen, Ludwigstraße 33, D-80539 München, Germany; 2 Sylvia Lawry Centre for Multiple Sclerosis Research, Hohenlindener Straße 1, D-81677 München, Germany; 3 Department of Statistics and Mathematics, Wirtschaftsuniversität Wien, Augasse 2 – 6, A-1090 Wien, Austria

## Abstract

**Background:**

Random forests are becoming increasingly popular in many scientific fields because they can cope with "small n large p" problems, complex interactions and even highly correlated predictor variables. Their variable importance measures have recently been suggested as screening tools for, e.g., gene expression studies. However, these variable importance measures show a bias towards correlated predictor variables.

**Results:**

We identify two mechanisms responsible for this finding: (i) A preference for the selection of correlated predictors in the tree building process and (ii) an additional advantage for correlated predictor variables induced by the unconditional permutation scheme that is employed in the computation of the variable importance measure. Based on these considerations we develop a new, conditional permutation scheme for the computation of the variable importance measure.

**Conclusion:**

The resulting conditional variable importance reflects the true impact of each predictor variable more reliably than the original marginal approach.

## 1 Background

Within the past few years, random forests [1] have become a popular and widely-used tool for non-parametric regression in many scientific areas. They show high predictive accuracy and are applicable even in high-dimensional problems with highly correlated variables, a situation which often occurs in bioinformatics. Recently, the variable importance measures yielded by random forests have also been suggested for the selection of relevant predictor variables in the analysis of microarray data, DNA sequencing and other applications [2-5].

Identifying relevant predictor variables, rather than only predicting the response by means of some "black-box" model, is of interest in many applications. By means of variable importance measures the candidate predictor variables can be compared with respect to their impact in predicting the response or even their causal effect (see, e.g., [6] for assumptions necessary for interpreting the importance of a variable as a causal effect). In this case a key advantage of random forest variable importance measures, as compared to univariate screening methods, is that they cover the impact of each predictor variable individually as well as in multivariate interactions with other predictor variables. For example, Lunetta et al. [2] find that genetic markers relevant in interactions with other markers or environmental variables can be detected more efficiently by means of random forests than by means of univariate screening methods like Fisher's exact test. In the analysis of amino acid sequence data Segal et al. [7] also point out the necessity to consider interactions between sequence positions. Tree-based methods like random forests can help identify relevant predictor variables even in such high dimensional settings involving complex interactions. Therefore, the impact of different amino acid properties, some of which have been shown to be relevant in DNA and protein evolution [8], for predicting peptide binding is investigated in our application example in Section 4. However, we will find in this application example, as often in practical problems, that many predictor variables are highly correlated.

The issue of correlated predictor variables is prominent in, but not limited to, applications in genomics and other high-dimensional problems. Therefore, it is important to note that in any non-experimental scientific study, where the predictor variable settings cannot be manipulated independently by the investigator, the distinction between the marginal and the conditional effect of a variable is crucial.

Consider, for example, the apparent correlation between rates of complication after surgery and mortality in hospitals, that was investigated by Silber and Rosenbaum [9]. It is plausible to believe that the mortality rate of a hospital depends on the rate of complications – or even that the mortalities are caused by the complications. However, when severity of illness is taken into account, the correlation disappears [9].

This phenomenon is known as a spurious correlation (see also Stigler [10] for a historical example). In the hospital mortality example, the spurious correlation is caused by the fact that hospitals that treat many serious cases have both higher complication and mortality rates. However, when conditioning on severity of illness (i.e. comparing only patients with similar severity of illness), mortality is no longer associated with complications.

If you consider this as a prediction problem, once the truly influential background variable (severity of illness) is known, it is clear that the remaining covariate (complication rate) provides no or little additional information for predicting the response (mortality rate). From a statistical point of view, however, this distinction can only be made by a conditional importance measure.

We will point out throughout this chapter that correlations between predictor variables – regardless of whether they arise from small-scale characteristics, such as proximities between genetic loci in organisms, or large-scale characteristics, such as similarities in the clientele of hospitals – severely affect the original random forest variable importance measures, because they can be considered as measures of marginal importance, even though what is of interest in most applications is the conditional effect of each variable. To make this distinction more clear, let us shortly review previous suggestions from the literature for measuring or illustrating variable importance in classification and regression trees (termed "classification trees" in the following for brevity, while all results apply to both classification and regression trees) and random forests: Breiman [11] displays the change in the response variable over the range of one predictor variable in "partial dependence plots" (see also [12] for a related approach). This may remind of the interpretation of model coefficients in linear models. However, whether the effect of a variable is interpretable as conditional on all other variables, as in linear models, may not be guaranteed in other models – and we will point out explicitly below that this is not the case in classification trees or random forests.

The permutation accuracy importance, that is described in more detail in Section 2.3, follows the rationale that a random permutation of the values of the predictor variable is supposed to mimic the absence of the variable from the model. The difference in the prediction accuracy before and after permuting the predictor variable, i.e. with and without the help of this predictor variable, is used as an importance measure. The actual permutation accuracy importance measure will be termed "permutation importance" in the following, while the general concept of the impact of a predictor variable in predicting the response is termed "variable importance". The alternative variable importance measure used in random forests, the Gini importance, is based on the principle of impurity reduction that is followed in most traditional classification tree algorithms. However, it has been shown to be biased when predictor variables vary in their number of categories or scale of measurement [13], because the underlying Gini gain splitting criterion is a biased estimator and can be affected by multiple testing effects [14]. Therefore, we will focus on the permutation importance in the following, that is reliable when subsampling without replacement – instead of bootstrap sampling – is used in the construction of the forest [13].

Based on the permutation importance, schemes for variable selection and for providing statements of the "significance" of a predictor variable (instead of a merely descriptive ranking of the variables w.r.t. their importance scores) have been derived: Breiman and Cutler [15] suggest a simple significance test that, however, shows poor statistical properties [16]. An approach for variable selection in large scale screening studies is introduced by Diaz-Uriarte and Alvarez de Andres [17], who suggest a backward elimination strategy. This approach has been shown to provide a reasonable selection of genes in many situations and is freely available in an R package [18], that also provides different plots for comparing the performance on the original data set to those on a data set with randomly permuted values of the response variable. The latter mimics the overall null hypothesis that none of the predictor variables is relevant and may serve as a baseline for significance statements. A similar approach is followed by Rodenburg et al. [19]. However, some recent simulation studies indicate that the performance of the variable importance measures may not be reliable when predictor variables are correlated: Even though Archer and Kimes [20] show in their extensive simulation study that the Gini importance can identify influential predictor variables out of sets of correlated covariates in many settings, the preliminary results of the simulation study of Nicodemus and Shugart [21] indicate that the ability of the permutation importance to detect influential predictor variables in sets of correlated covariates is less reliable than that of alternative machine learning methods and highly depends on the number of previously selected splitting variables mtry. These studies, as well as our simulation results, indicate that random forests show a preference for correlated predictor variables, that is also carried forward to any significance test or variable selection scheme constructed from the importance measures.

In this work we aim at providing a deeper understanding of the underlying mechanisms responsible for the observations of [20] and [21]. In addition to this, we want to broaden the scope of considered problems to the comparison of the influence of correlated and uncorrelated predictor variables. For this type of problem we introduce a new, conditional permutation importance for random forests, that better reflects the true importance of predictor variables. Our approach is motivated by the visual means of illustration introduced by Nason et al. [22]: In their "CARTscans" plots they not only display the marginal influence of a predictor variable, like the partial dependence plots of Breiman [11], but the influence of continuous predictor variables separately for the levels of two other, categorical predictor variables, namely a conditional influence plot.

As pointed out above, in the case of correlated predictor variables it is important to distinguish between conditional and marginal influence of a variable, because a variable that may appear influential marginally might actually be independent of the response when considered conditional on another variable. In this respect the approach of [22] is an important improvement, but in its current form is only applicable for categorical covariates. Therefore our aim in this work is to provide a general scheme that can be used both for illustrating the effect of a variable and for computing its permutation importance conditional on relevant covariates of any type. While the conditioning scheme of [22] can be considered as a full-factorial cross-tabulation based on two categorical predictor variables, our conditioning scheme is based on a partition of the entire feature space that is determined directly by the fitted random forest model.

In the following Section 2 we will outline how ensembles of classification trees are constructed and illustrate in a simulation study why correlated predictor variables tend to be overselected. Then we will review the construction of the original permutation importance before we introduce a new permutation scheme that we suggest for the construction of a conditional permutation importance measure. The advantage of this measure over the currently-used one is illustrated in the results of our simulation study in Section 3 and in the application to peptide-binding data in Section 4.

## 2 Methods

In random forests and the related method bagging, an ensemble of classification trees is created by means of drawing several bootstrap samples or subsamples from the original training data and fitting a single classification tree to each sample. Due to the random variation in the samples and the instability of the single classification trees, the ensemble will consist of a diverse set of trees. For prediction, a vote (or average) over the predictions of the single trees is used and has been shown to highly outperform the single trees: By combining the prediction of a diverse set of trees, bagging utilizes the fact that classification trees are instable but on average produce the right prediction. This understanding has been supported by several empirical studies (see, e.g., [23-26]) and especially the theoretical results of Bühlmann and Yu [27], who could show that the improvement in the prediction accuracy of ensembles is achieved by means of smoothing the hard cut decision boundaries created by splitting in single classification trees, which in return reduces the variance of the prediction.

In random forests, another source of diversity is introduced when the set of predictor variables to select from is randomly restricted in each split, producing even more diverse trees. In addition to the smoothing of hard decision boundaries, the random selection of splitting variables in random forests allows predictor variables that were otherwise outplayed by their competitors to enter the ensemble. Even though these variables may not be optimal with respect to the current split, their selection may reveal interaction effects with other variables that otherwise would have been missed and thus work towards the global optimality of the ensemble.

The classification trees, from which the random forests are built, are built recursively in that the next splitting variable is selected by means of locally optimizing a criterion (such as the Gini gain in the traditional CART algorithm [28]) within the current node. This current node is defined by a configuration of predictor values, that is determined by all previous splits in the same branch of the tree (see, e.g., [29] for illustrations). In this respect the evaluation of the next splitting variable can be considered conditional on the previously selected predictor variables, but regardless of any other predictor variable. In particular, the selection of the first splitting variable involves only the marginal, univariate association between that predictor variable and the response, regardless of all other predictor variables. However, this search strategy leads to a variable selection pattern where a predictor variable that is per se only weakly or not at all associated with the response, but is highly correlated with another influential predictor variable, may appear equally well suited for splitting as the truly influential predictor variable. We will illustrate this point in more detail in the following simulation study.

### 2.1 Simulation design

A simulation study was set up in order to illustrate the treatment of correlated predictor variables in ensemble methods based on classification trees. Data sets were generated according to a linear model with twelve predictor variables *y*_*i *_= *β*_1_·*x*_*i*,1 _+ ⋯ + *β*_12_·*x*_*i*,12 _+ *ε*_*i*_, with $\varepsilon_{i}\overset{i.i.d.}{\underset{}{\sim }}N(0,0.5)$. The predictor variables were sampled from a multivariate normal distribution *X*_1_,..., *X*_12 _~ *N*(0, **Σ**) where the covariance structure **Σ **was chosen such that all variables have unit variance *σ*_*j*, *j *_= 1 and only the first four predictor variables are block-correlated with *σ*_*j*, *j' *_= 0.9 for *j *≠ *j' *≤ 4, while the rest were independent with *σ*_*j*, *j' *_= 0. Of the twelve predictor variables only six were influential, as indicated by their coefficients in Table 1. A covariance structure of this type was already used for illustrating the effect of correlations by Archer and Kimes [20]. However, while their study mainly aimed at identifying one influential predictor out of a correlated set, here we also want to compare the importance scores of predictor variables with equally large coefficients, while some of the predictor variables are correlated and others are not: *X*_1_,..., *X*_4 _and *X*_5_,..., *X*_8 _share the same coefficient pattern, while only *X*_1_,..., *X*_4 _are correlated. From the generated data sets, random forests were built with the cforest function from the party package [30,31] in the R system for statistical computing [32]. Different values for the parameter mtry, that regulates the number of randomly preselected splitting variables, were considered to be able to investigate the mechanisms responsible for the results of Nicodemus and Shugart [21]. Default settings were used for all other parameters.

**Table 1:** Simulation design. Regression coefficients of the data generating process.

*X*_*j*_	*X*_1_	*X*_2_	*X*_3_	*X*_4_	*X*_5_	*X*_6_	*X*_7_	*X*_8_	⋯	*X*_12_
*β*_*j*_	5	5	2	0	-5	-5	-2	0	⋯	0

### 2.2 Illustration of variable selection

**Figure 1:**
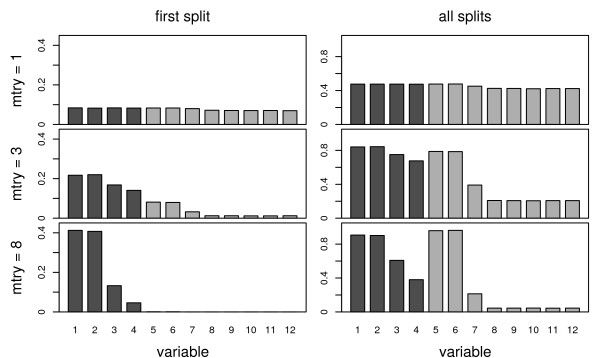
**Selection rates**. Relative selection rates for twelve variables in the first splits (left) and in all splits (right) of all trees in random forests built with different values for mtry.

We find in the panel on the left hand side of Figure 1 that in the first splits of all trees, where the variables are considered only marginally with respect to their association to the response, those variables (*X*_3 _and *X*_4_) correlated with highly influential predictors are selected equally often as the highly influential predictor variables (*X*_1 _and *X*_2 _as well as *X*_5 _and *X*_6_) for mtry = 1, where no competitors are available and the correlated predictors can serve as replacements of the influential ones (the fact that the non-influential predictor variables *X*_8 _through *X*_12 _are selected almost equally often is only due to the lax choice of the stop criterion). When mtry increases and the highly influential variables may be available as predominant competitors in some splits those variables (*X*_3 _and *X*_4_) correlated with highly influential predictors are selected less often than the highly influential correlated ones (*X*_1 _and *X*_2_) themselves, but more often than even the highly influential uncorrelated ones (*X*_5 _and *X*_6_). When we consider all splits of all trees in the panel on the right hand side of Figure 1, the correlated predictors loose most of their advantage because variable selection is now conditional on the previously chosen variables in the same branch of the tree, that may include the truly influential correlated predictors. However, since variable selection is not conditional on all (or at least all correlated) variables, there is still a preference for the correlated variables with low and zero coefficients (*X*_3 _and *X*_4 _over *X*_7 _and *X*_8_), with a similar dependency on mtry.

This selection pattern is due to the locally optimal variable selection scheme used in recursive partitioning, that considers only one variable at a time and conditional only on the current branch. However, since this characteristic of tree-based methods is a crucial means of reducing computational complexity (and any attempts to produce globally optimal partitions are strictly limited to low dimensional problems at the moment [33]), it shall remain untouched here.

### 2.3 The permutation importance

The rationale of the original random forest permutation importance is the following: By randomly permuting the predictor variable *X*_*j*_, its original association with the response *Y *is broken. When the permuted variable *X*_*j*_, together with the remaining non-permuted predictor variables, is used to predict the response for the out-of-bag observations, the prediction accuracy (i.e. the number of observations classified correctly) decreases substantially if the original variable *X*_*j *_was associated with the response. Thus, Breiman [1] suggests the difference in prediction accuracy before and after permuting *X*_*j*_, averaged over all trees, as a measure for variable importance, that we formalize as follows: Let ${\bar{ℬ}}^{\left(t\right)}$ be the out-of-bag (oob) sample for a tree *t*, with *t *∈ {1,..., *ntree*}. Then the variable importance of variable *X*_*j *_in tree *t *is

(1) $$VI^{\left(t\right)}(X_{j})=\frac{\sum_{i\in {\bar{ℬ}}^{\left(t\right)}}I\left(y_{i}={\hat{y}}_{i}^{\left(t\right)}\right)}{\left|{\bar{ℬ}}^{\left(t\right)}\right|}-\frac{\sum_{i\in {\bar{ℬ}}^{\left(t\right)}}I\left(y_{i}={\hat{y}}_{i,\pi_{j}}^{\left(t\right)}\right)}{\left|{\bar{ℬ}}^{\left(t\right)}\right|}$$

where ${\hat{y}}_{i}^{\left(t\right)}=f^{\left(t\right)}(x_{i})$ is the predicted class for observation *i *before and ${\hat{y}}_{i,\pi_{j}}^{\left(t\right)}=f^{\left(t\right)}(x_{i,\pi_{j}})$ is the predicted class for observation *i *after permuting its value of variable *X*_*j*_, i.e. with $x_{i,\pi_{j}}=(x_{i,1},...,x_{i,j-1,}x_{\pi_{j}(i),j},x_{i,j+1},...,x_{i,p})$. (Note that *VI*^(*t*)^(**X**_*j*_) = 0 by definition, if variable *X*_*j *_is not in tree *t*.) The raw variable importance score for each variable is then computed as the mean importance over all trees: $VI(X_{j})=\frac{\sum_{t=1}^{ntree}VI^{\left(t\right)}(x_{j})}{ntree}$

In standard implementations of random forests an additional scaled version of the permutation importance (often called *z*-score), that is achieved by dividing the raw importance by its standard error, is provided. However, since recent results [16,17] indicate that the raw importance *VI*(**X**_*j*_) has better statistical properties, we will only consider the unscaled version here.

### 2.4 Types of independence

We know that the original permutation importance overestimates the importance of correlated predictor variables. Part of this artefact may be due to the preference of correlated predictor variables in early splits as illustrated in Section 2.2. However, we also have to take into account the permutation scheme that is employed in the computation of the permutation importance. In the following we will first outline what notion of independence corresponds to the current permutation scheme of the random forest permutation importance. Then we will introduce a more sensible permutation scheme that better reflects the true impact of predictor variables.

It can help our understanding to consider the permutation scheme in the context of permutation tests [34]: Usually a null hypothesis is considered that implies the independence of particular (sets of) variables. Under this null hypothesis some permutations of the data are permitted because they preserve the structure determined by the null hypothesis. If, for example, the response variable *Y *is independent from all predictor variables (global null hypothesis) a permutation of the (observed) values of *Y *affects neither the marginal distribution of *Y *nor the joint distribution of *X*_1_,..., *X*_*p *_and *Y*, because the joint distribution can be factorized as *P*(*Y*, *X*_1_,..., *X*_*p*_) = *P*(*Y*)·*P*(*X*_1_,..., *X*_*p*_) under the null hypothesis. If, however, the null hypothesis is not true, the same permutation will lead to a deviation in the joint distribution or some reasonable test statistic computed from it. Therefore, a change in the distribution or test statistic caused by the permutation can serve as an indicator that the data do not follow the independence structure we would expect under the null hypothesis.

With this framework in mind, we can now take a second look at the random forest permutation importance and ask: Under which null hypothesis would this permutation scheme be permitted? If the data are actually generated under this null hypothesis the permutation importance will be (a random value from a distribution with mean) zero, while any deviation from the null hypothesis will lead to a change in the prediction accuracy, that is used as a test statistic here, and thus will be detectable as an increase in the value of the permutation importance.

**Figure 2:**
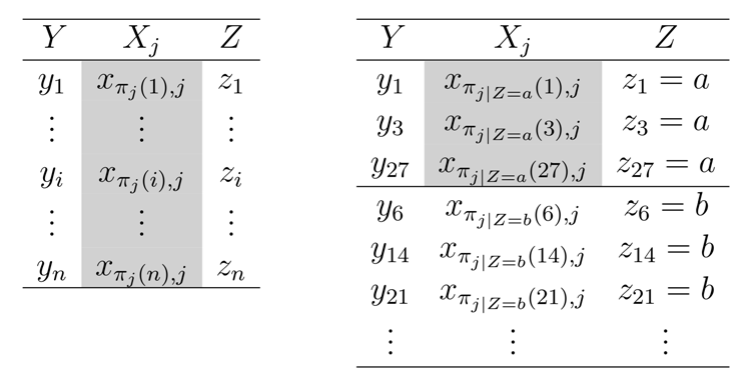
Permutation scheme for the original marginal (left) and for the newly suggested conditional (right) permutation importance.

We find that the original permutation importance, where one predictor variable *X*_*j *_is permuted against both the response *Y *and the remaining (one or more) predictor variables *Z *= *X*_1_,..., *X*_*j*-1_, *X*_*j*+1_,..., *X*_*p *_as illustrated in the left panel of Figure 2, corresponds to a null hypothesis of independence between *X*_*j *_and both *Y *and *Z*:

(2) *H*_0 _: *X*_*j *_⊥ *Y*, *Z *or equivalently *X*_*j *_⊥ *Y *∧ *X*_*j *_⊥ *Z*

Under this null hypothesis the joint distribution can be factorized as

(3) $$P(Y,X_{j},Z)\overset{H_{0}}{\underset{}{=}}P(Y,Z)\cdot P(X_{j}).$$

What is crucial when we want to understand why correlated predictor variables are preferred by the original random forest permutation importance is that a positive value of the importance corresponds to a deviation from this null hypothesis – that can be caused by a violation of either part: the independence of *X*_*j *_and *Y*, or the independence of *X*_*j *_and *Z*. However, from these two aspects only one is of interest when we want to assess the impact of *X*_*j *_to help predict *Y*, namely the question if *X*_*j *_and *Y *are independent. This aim, to measure only the impact of *X*_*j *_on *Y*, would be better reflected if we could create a measure of deviation from the null hypothesis that *X*_*j *_and *Y *are independent under a given correlation structure between *X*_*j *_and the other predictor variables, that is determined by our data set. To meet this aim we suggest a conditional permutation scheme, where *X*_*j *_is permuted only within groups of observations with *Z *= *z*, to preserve the correlation structure between *X*_*j *_and the other predictor variables as illustrated in the right panel of Figure 2.

This permutation scheme corresponds to the following null hypothesis

(4) *H*_0 _: (*X*_*j *_⊥ *Y*)|*Z*,

where the conditional distribution can be factorized under the null hypothesis as

(5) $$\begin{array}{llll} & P(Y,X_{j}|Z) & \overset{H_{0}}{\underset{}{=}} & P(Y|Z)\cdot P(X_{j}|Z)\\ \text{or} & P(Y|X_{j},Z) & \overset{H_{0}}{\underset{}{=}} & P(Y|Z), \end{array}$$

which is the definition of conditional independence.

In the special case where *X*_*j *_and *Z *are independent both permutation schemes will give the same result, as illustrated by our simulation results below. When *X*_*j *_and *Z *are correlated, however, the original permutation scheme will lead to an apparent increase in the importance of correlated predictor variables, that is due to deviations from the uninteresting null hypothesis of independence between *X*_*j *_and *Z*.

### 2.5 A new, conditional permutation scheme

Technically, any kind of conditional assessment of the importance of one variable conditional on another one is straightforward whenever the variables to be conditioned on, *Z*, are categorical as in [22]. However, for our aim to conditionally permute the values of *X*_*j *_within groups of *Z *= *z*, where *Z *can contain potentially large sets of covariates of different scales of measurement, we want to supply a grid that (i) is applicable to variables of different types, (ii) is as parsimonious as possible, but (iii) is also computationally feasible. Our suggestion is to define the grid within which the values of *X*_*j *_are permuted for each tree by means of the partition of the feature space induced by that tree. The main advantages of this approach are that this partition was already learned from the data during model fitting, contains splits in categorical, ordered and continuous predictor variables and can thus serve as an internally available means for discretizing the feature space.

In principle, any partition derived from a classification tree can be used to define the permutation grid. Here we used partitions produced by unbiased conditional inference trees [31], that employ binary splitting as in the standard CART algorithm [28]. This means that, if *k *is the number of categories of an unordered or ordered categorical variable, up to *k*, but potentially less than *k*, subsets of the data are separated.

Continuous variables are treated in the same way: Every binary split in a variable provides one or more cutpoints, that can induce a more or less fine graded grid on this variable. By using the grid resulting from the current tree we are able to condition in a straightforward way not only on categorical, but also on continuous variables and create a grid that may be more parsimonious than the full factorial approach of [22]. Only in one aspect we suggest to leave the recursive partition induced by a tree: Within a tree structure, each cutpoint refers to a split in a variable only within the current node (i.e. a split in a variable may not bisect the entire sample space but only partial planes of it). However, for ease of computation, we suggest that the conditional permutation grid uses all cutpoints as bisectors of the sample space (the same approach is followed by [22]). This leads to a more fine graded grid, and may in some cases result in small cell frequencies inducing greater variation (even though our simulation results indicate that in practice this is not a critical issue). From a theoretical point of view, however, conditioning too strictly has no negative effect, while a lack of conditioning produces artefacts as observed for the unconditional permutation importance.

In summary the conditional permutation importance is derived as follows:

1. In each tree compute the oob-prediction accuracy before the permutation as in Equation 1: $\frac{\sum_{i\in {\bar{ℬ}}^{\left(t\right)}}I\left(y_{i}={\hat{y}}_{i}^{\left(t\right)}\right)}{\left|{\bar{ℬ}}^{\left(t\right)}\right|}$.

2. For all variables *Z *to be conditioned on: Extract the cutpoints that split this variable in the current tree and create a grid by means of bisecting the sample space in each cutpoint.

3. Within this grid permute the values of *X*_*j *_and compute the oob-prediction accuracy after permutation: $\frac{\sum_{i\in {\bar{ℬ}}^{\left(t\right)}}I\left(y_{i}={\hat{y}}_{i,\pi_{j}|Z}^{\left(t\right)}\right)}{\left|{\bar{ℬ}}^{\left(t\right)}\right|}$, where ${\hat{y}}_{i,\pi_{j}|Z}^{\left(t\right)}=f^{\left(t\right)}(x_{i,\pi_{j}|Z})$ is the predicted classes for observation *i *after permuting its value of variable *X*_*j *_within the grid defined by the variables *Z*.

4. The difference between the prediction accuracy before and after the permutation accuracy again gives the importance of *X*_*j *_for one tree (see Equation 1). The importance of *X*_*j *_for the forest is again computed as an average over all trees.

To determine the variables *Z *to be conditioned on, the most conservative – or rather overcautious -strategy would be to include all other variables as conditioning variables, as was indicated by our initial notation. A more intuitive choice is to include only those variables whose empirical correlation with the variable of interest *X*_*j *_exceeds a certain moderate threshold, as we do with the Pearson correlation coefficient for continuous variables in the following simulation study and application example. For the more general case of predictor variables of different scales of measurement the framework promoted by Hothorn et al. [31] provides p-values of conditional inference tests as measures of association. The p-values have the advantage that they are comparable for variables of all types and can serve as an intuitive and objective means for selecting the variables *Z *to be conditioned on in any problem. Another option is to let the user himself select certain variables to condition on, if, e.g., a hypothesis of interest includes certain independencies.

Note however, that neither a high number of conditioning variables nor a high overall number of variables in the data set poses a problem for the conditional permutation approach: The permutation importance is computed individually for each tree and then averaged over all trees. Correspondingly, the conditioning grid for each tree is determined by the partition of that particular tree only. Thus, even if in principle the stability of the permutation may be affected by small cell counts in the grid, practically the complexity of the grid is limited by the depth of each tree.

The depth of the tree, however, does not depend on the overall number of predictor variables, but on various other characteristics of the data set (most importantly the ratio of relevant vs. noise variables, that is usually low, for example in genomics) in combination with tuning parameter settings (including the number of randomly preselected predictor variables, the split selection criterion, the use of stopping criteria and so forth). Lin and Jeon [35] even point out that limiting the depth of the trees in random forests may prove beneficial w.r.t. prediction accuracy in certain situations.

Another important aspect is that the conditioning variables, especially if there are many, may not necessarily appear all together with the variable of interest in each individual tree, but different combinations may be represented in different trees if the forest is large enough.

## 3 Results

**Figure 3:**
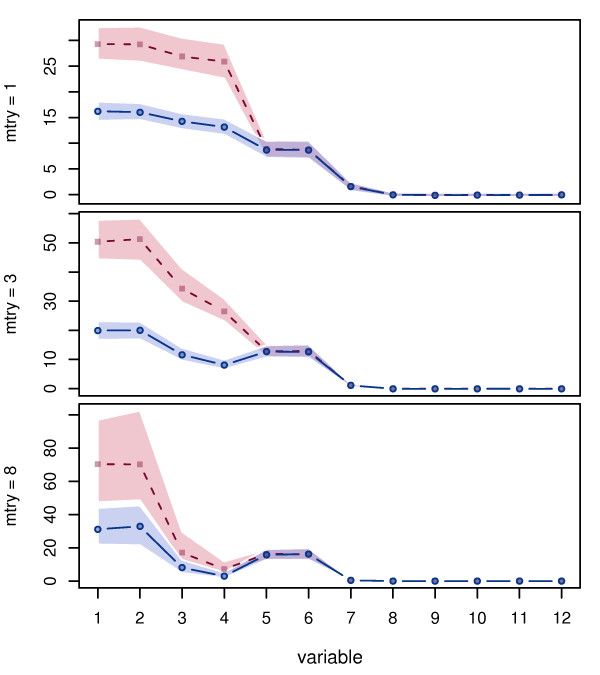
**Permutation importance**. Median permutation importance for marginal (dashed) and conditional (solid) permutation scheme along with inter-quartile range. Note that the ordering of variables in the plot is arbitrary.

For the simulation design introduced in Section 2.1, Figure 3 shows the median and interquartile range (over 500 iterations) of the importance scores of each variable for the different permutation schemes: the original marginal permutation and the newly suggested conditional permutation scheme. The set of variables *Z *to be conditioned on was chosen here to include all variables with an empirical correlation *r *≥ .2.

We find that the pattern of the coefficients induced in the data generating process is not reflected by the importance values computed with the ordinary permutation scheme. With this scheme the importance scores of the correlated predictor variables are highly overestimated. This effect is most pronounced for small values of mtry, because correlated variables have a higher chance to end up in a top position in a tree when their correlated competitors are not available.

For the conditional permutation scheme the importance scores better reflect the true pattern: The correlated variables *X*_1 _and *X*_2 _with the same coefficient show an almost equal level of importance as the uncorrelated variables *X*_5 _and *X*_6_, while the importance of *X*_3 _and *X*_4_, that are correlated but have a lower or zero coefficient, decrease. For the variables with small and zero coefficients we still find a difference between the correlated and uncorrelated variables, such that for the correlated variables the importance values are still overestimated – however to a much lesser extent than with the unconditional permutation scheme. This remaining disadvantage of the uncorrelated predictor variables may be due to the fact that for most values of mtry these variables are selected less often and in lower positions in the tree (see Figure 1) and thus have a lower chance to produce a high importance value. The degree of the preference of correlated predictor variables also depends on the choice of mtry and is most pronounced for small values of mtry, as expected from the selection frequencies. On the other hand, we find in Figure 3 that the variability of the importance increases for large values of mtry, and the prediction accuracy is expected to be higher for smaller values of mtry. Another interesting feature of the conditional permutation scheme is that the variability of the conditional importance is lower than that of the unconditional importance within each level of mtry.

With respect to the identifiability of few influential predictors from a set of correlated and other noise variables (which was the task in [20] and [21]), we can see from the importance scores for *X*_1_,..., *X*_3 _in comparison to that of *X*_4 _that the conditional importance reflects the same pattern as the unconditional importance, however with a notably smaller variation that may improve the identifiability. In the comparison of potentially influential correlated and uncorrelated predictor variables on the other hand, the conditional importance is much better suited as a means of comparison than the original importance. For piecewise constant functions, that can be more easily addressed with recursive partitioning methods, the beneficial effect of conditioning is even stronger than presented here.

## 4 Example: Relating amino acid sequence to phenotype in peptide-binding data

**Figure 4:**
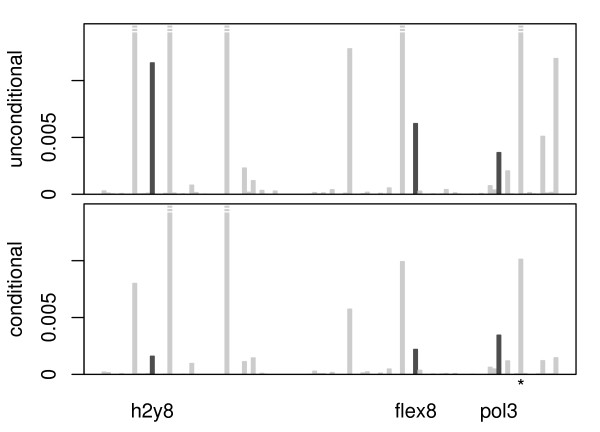
**Example: peptide-binding data**. Marginal (top) and conditional (bottom) permutation importance of 104 predictors of peptide-binding.

As an application example we consider peptide-binding data that were previously analysed with recursive partitioning techniques by Segal et al. [7]. The data set includes 105 variables for a total of *n *= 310 amino acid sequences. The response to be predicted is a binding property that can be coded as a binary variable (binding/no binding). The remaining variables available in this data set correspond to 13 amino acid properties for each of the eight considered amino acid positions. These 13 properties include, e.g. volume, polarity, bulkiness, flexibility, aromaticity, and charge, yielding in total 104 continuous predictor variables. A random forest with 1000 trees and mtry = 104 (which corresponds to bagging [23,24] as a special case of a random forest where mtry is equal to the number of candidate predictors and variable selection is not randomly restricted) was fit to the data set. The permutation importance was computed either with the unconditional or the conditional permutation scheme. The resulting importance scores are displayed in Figure 4 (note that the absolute values of the scores should not be interpreted). The few predictor variables whose importance scores reach highest or even exceed the plotting area would be selected for further analysis by any means. However, for some of the variables with the next smaller importance scores the ranking strongly depends on the permutation scheme. We will focus our illustration on the ranking of three exemplary predictor variables, "h2y8", "flex8" and "pol3", that are highlighted in Figure 4: We find in the unconditional view in the top panel of Figure 4 that "h2y8" and "flex8" appear to be of higher importance than "pol3" (ranks "h2y8": 8, "flex8": 9, "pol3": 11). However, in the conditional view in the bottom panel of Figure 4 their order is reversed and it turns out that "pol3" is really more important than "h2y8" and "flex8"(ranks "h2y8": 9, "flex8": 8, "pol3": 7). This change in the ranks of the predictor variables is most pronounced for large mtry as expected, but similar effects can be observed for smaller values.

When exploring the reason why the importances of "h2y8" and "flex8" are moderated by conditioning, while the importance of "pol3" remains almost constant, we find that "h2y8" and "flex8" are correlated with influential covariates, while "pol3" is only correlated with non-influential covariates. For example, "h2y8" is highly correlated with the polarity at position eight "pol8", that is indicated by the * symbol in in Figure 4. The variable "pol8" shows a high importance (that is however also moderated by conditioning) and was already found to be influential by Segal et al. [7], who note that it may approximate an effect of the eighth position in the original sequence data, while the results of Xia and Li [8] indicate an effect of the amino acid property polarity itself.

This shows that importance rankings in data sets that contain complex correlations between predictor variables can be severely affected by the underlying permutation scheme: When the conditional permutation is used, the importance scores of correlated predictor are moderated such that the truly influential predictor variables have a higher chance to be detected.

## 5 Discussion and conclusion

We have investigated the sources of preferences in the variable importance measures of random forests in favor of correlated predictor variables and suggested a new, conditional permutation scheme for the computation of the variable importance measure. This new, conditional permutation scheme uses the partition that is automatically provided by the fitted model as a conditioning grid and reflects the true impact of each predictor variable better than the original, marginal approach. Even though the conditional permutation cannot entirely eliminate the preference for correlated predictor variables, it has been shown to provide a more fair means of comparison that can help identify the truly relevant predictor variables. Our simulation results also illustrate the impact of the choice of the random forest tuning parameter mtry: While the default value mtry = $\sqrt{p}$ is often found to be optimal with respect to prediction accuracy in empirical studies [36], our findings indicate that in the case of correlated predictor variables different values of mtry should be considered. However, it should also be noted that any interpretation of random forest variable importance scores can only be sensible when the number of trees is chosen sufficiently large such that the results produced with different random seeds do not vary systematically. Only then it is assured that the differences between, e.g., unconditional and conditional importance are not only due to random variation.

The conditional permutation importance will be freely available in the next release of the party package for recursive partitioning [30,31] in the R system for statistical computing [32].

## Authors' contributions

CS defined the research question, suggested the conditional variable importance, set up and performed the simulation experiments and drafted the manuscript. A–LB analyzed the peptide-binding data. TK, TA and AZ contributed to the theoretical understanding and presentation of the problem. All authors contributed to and approved the final version of the manuscript.
